# Independent effects of sex and stress on fructose‐induced salt‐sensitive hypertension

**DOI:** 10.14814/phy2.15489

**Published:** 2022-10-06

**Authors:** Autumn Brostek, Nancy J. Hong, Ronghao Zhang, Beau R. Forester, Lauren E. Barmore, Lindsey Kaydo, Nicholas Kluge, Corey Smith, Jeffrey L. Garvin, Agustin Gonzalez‐Vicente

**Affiliations:** ^1^ Department of Physiology and Biophysics Case Western Reserve University School of Medicine Cleveland Ohio USA; ^2^ Department of Nephrology and Hypertension Cleveland Clinic Glickman Urological & Kidney Institute Cleveland Ohio USA

**Keywords:** blood pressure, norepinephrine, renal sympathetic nerve, salt‐sensitive hypertension

## Abstract

Proximal tubule fructose metabolism is key to fructose‐induced hypertension, but the roles of sex and stress are unclear. We hypothesized that females are resistant to the salt‐sensitive hypertension caused by low amounts of dietary fructose compared to males and that the magnitude of the increase in blood pressure (BP) depends, in part, on amplification of the stress response of renal sympathetic nerves. We measured systolic BP (SBP) in rats fed high salt with either no sugar (HS), 20% glucose (GHS) or 20% fructose (FHS) in the drinking water for 7–8 days. FHS increased SBP in both males (Δ22 ± 9 mmHg; *p* < 0.046) and females (Δ16 ± 3 mmHg; *p* < 0.0007), while neither GHS nor HS alone induced changes in SBP in either sex. The FHS‐induced increase in SBP as measured by telemetry in the absence of added stress (8 ± 2 mmHg) was significantly lower than that measured by plethysmography (24 ± 5 mmHg) (*p* < 0.014). However, when BP was measured by telemetry simulating the stress of plethysmography, the increase in SBP was significantly greater (15 ± 3 mmHg) than under low stress (8 ± 1 mmHg) (*p* < 0.014). Moderate‐stress also increased telemetric diastolic (*p* < 0.006) and mean BP (*p* < 0.006) compared to low‐stress in FHS‐fed animals. Norepinephrine excretion was greater in FHS‐fed rats than HS‐fed animals (Male: 6.4 ± 1.7 vs.1.8 ± 0.4 nmole/kg/day; *p* < 0.02. Female 54 ± 18 vs. 1.2 ± 0.6; *p* < 0.02). We conclude that fructose‐induced salt‐sensitive hypertension is similar in males and females unlike other forms of hypertension, and the increase in blood pressure depends in part on an augmented response of the sympathetic nervous system to stress.

## Graphical abstract

We show that rats consuming moderate amounts of fructose in combination with elevated dietary sodium present an increased renal sympathetic output and develop hypertension. Adding moderate stress as an independent variable increases systolic blood pressure even further, and in a similar magnitude in males and females.Fructose‐induced salt‐sensitive hypertension depends on renal sympathetic output in males and females

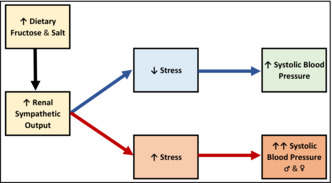

## 1 INTRODUCTION

Dietary fructose consumption in the United States is currently nearly 20‐fold greater than it was in 1970 (Hallfrisch, 1990; Marriott et al., 2009). Clinical and basic science studies have linked the consumption of fructose, primarily as high fructose corn syrup, to many pathologies including kidney failure (Gersch et al., 2007; Nakayama et al., 2010; Sanchez‐Lozada et al., 2007) and hypertension (Hwang et al., 1987; Jalal et al., 2010; Nguyen et al., 2009), especially salt‐sensitive hypertension (Brown et al., 2011; Nishimoto et al., 2002; Sechi, 1999). The last of these is likely exacerbated by the fact that salt consumption has also increased by about 50% over this period (Briefel & Johnson, 2004).

Blood pressure per se and the incidence of hypertension are well known to depend on sex. Men generally have higher blood pressures than women (Mozaffarian et al., 2016) and men are also more likely than women to develop hypertension (Mills et al., 2020). Fructose‐induced salt‐sensitive hypertension has been reported to depend on either an activated renin‐angiotensin‐aldosterone system (RAS) or exaggerated responses to angiotensin II (Farah et al., 2007; Gonzalez‐Vicente et al., 2017, 2018; Navarro‐Cid et al., 1995). Males are significantly more sensitive to the hypertensive actions of angiotensin II than females (Brown et al., 2012; Gillis & Sullivan, 2016; Sampson et al., 2008; Shukri et al., 2018; Xue et al., 2005; Zimmerman & Sullivan, 2013). However, whether fructose‐induced salt‐sensitive hypertension is greater in males than females is unknown.

The effects of low to moderate amounts of dietary fructose on blood pressure are somewhat controversial in experimental models because the blood pressure response to fructose is variable (Madero et al., 2011), with increases ranging from 4 (Abdulla et al., 2012) to greater than 20 mmHg (Di Verniero et al., 2008; El‐Bassossy & Shaltout, 2015). The explanation for this is unclear but it may largely depend on differences in sympathetic nerve activation. The effects of dietary fructose on blood pressure are known to involve sympathetic nerve activity (Di Verniero et al., 2008; Komnenov et al., 2019; Soncrant et al., 2018; Valensi, 2005; Verma et al., 1999) and differences in sympathetic activity have been proposed as an explanation for the disparate results of the effects of fructose on blood pressure (Madero et al., 2011).

Variability in sympathetic nerve activation may result from differences in the method of blood pressure measurement and/or environmental stressors between studies. Previously, it was shown that careful measurement of blood pressure by tethered catheters yielded larger increases in blood pressure than those measured by telemetry. Given that in both methods, blood pressure was measured via catheters placed in the aorta, the authors concluded that the differences were caused by the additional stress, and thus sympathetic activation, caused by the tethered catheters themselves (King et al., 2006). Despite these conclusions, the effect of stress per se on fructose‐induced salt‐sensitive hypertension has not been directly investigated. Also, the combined effects of stress and dietary fructose on urinary norepinephrine excretion, a measure of sympathetic tone, remains unknown.

We hypothesized that females are resistant to the salt‐sensitive hypertension caused by low amounts of dietary fructose compared to males and that the magnitude of the increase in blood pressure depends, in part, on amplification of the stress response of renal sympathetic nerves.

## 2 METHODS

### 2.1 Animals

All protocols involving animals were approved by the Case Western Reserve University Institutional Animal Care and Use Committee. Sprague–Dawley rats were purchased from Charles River breeding laboratories. Rats were fed a standard rodent diet (Prolab Isopro RMH 3000) for basal measurements upon arrival in the animal care facility. This diet contained 0.70% NaCl, 5.0% fiber, 30.4% starch and 60% of caloric content from carbohydrates. After baseline measurements were completed, the standard rodent diet was switched to a high‐salt diet (4% NaCl; TestDiet #9GDV) with either tap water (high salt alone), 20% glucose (glucose/high salt) or 20% fructose (fructose/high salt) in the drinking water for 7–8 days. Test Diet #9GDV contained 3.67% NaCl, 4.3% fiber, 15% sucrose, and 55.6% of caloric content coming from carbohydrates. Male and female 6‐ to 10‐week‐old rats were used for this study. Stage of estrus cycle was not considered in female rats.

### 2.2 Blood pressure by plethysmography

Systolic blood pressure was recorded by tail plethysmography using a CODA Non‐invasive Blood Pressure System (Kent Scientific Corporation) in male and female rats using methods similar to those we used previously (Gonzalez‐Vicente et al., 2017; Hong et al., 2021). Rats were trained three times the week prior to the beginning of the experiment while on a standard rodent diet. For this, animals were moved on a cart from the housing room to the plethysmography equipment room where blood pressure was measured, and returned immediately after training was complete. Measurements were made in a “quiet” room used only for plethysmography blood pressure measurements for this study. No other investigators used this room for any purpose. Training consisted of placing rats in the restraint used for actual measurements, putting the plethysmography cuff on the tail, and laying rats on a heating pad for 10–15 min. The cuff was inflated as it would be during actual experiments but the data were not recorded. After 2–3 training sessions and a baseline measurement, animals were randomly split into different groups and the standard rodent diet was changed to either high salt alone, glucose/high salt or fructose/high salt. Then systolic blood pressure was measured and recorded every 2–3 days for an additional 7–8 days. Changes in systolic blood pressure were calculated by subtracting the baseline systolic blood pressure on day 0 from the systolic blood pressure at the end of the experiment.

### 2.3 Blood pressure by telemetry

Systolic blood pressure was measured by telemetry in male rats as we have previously reported (Cabral et al., 2014). Briefly, rats were anesthetized with isoflurane (4% induction and 2% maintenance) and an incision was made in the left leg to expose the femoral artery. A catheter was placed in the artery and threaded to the aorta. A pocket was made under the skin on the left flank and the telemetry probe (Data Sciences International, Model HDX‐10) was inserted. The wound was closed and rats were allowed to recover for 3 days prior to the beginning of the experiment.

Blood pressure was measured by telemetry using two different protocols considered “low stress” and “moderate stress”. For “moderate stress”, we used a protocol that mimicked that used to measure blood pressure by plethysmography. In brief, rats were housed in the general rat room with the animals of other investigators. To measure blood pressure, rats were moved on a cart from the rat housing room to a quiet room used to measure blood pressure by telemetry. Animals were trained for 2 consecutive days and baseline blood pressure was recorded on the 3rd day. Then the diet was changed from a standard rodent diet to fructose/high salt, and blood pressure recorded every other day for 7–8 days. After blood pressure was measured, they were returned to the general housing room. Due to the transport of rats between two different rooms, the animals in this protocol were considered to be under “moderate stress”.

In the “low stress” protocol, systolic blood pressures were measured in the same room where rats were housed. Thus, a key difference with the “moderate stress” is that animals were not moved on a cart before and after measurements; thus, systolic blood pressure was measured without disturbing the animals. In addition, to test whether there was an effect of the room in which the rats were housed (general housing vs. dedicated room) rats were either housed in the general rat room or in the room in which systolic blood pressure was measured under the “moderate stress” protocol. Basal blood pressure was recorded on 2 consecutive days. Then diets were changed to fructose/high salt, and blood pressure was recorded every other day for 7–8 days. Both basal and fructose‐induced increases in systolic blood pressure were similar in all “low stress” rats regardless of housing location, so the results were pooled.

### 2.4 Urinary norepinephrine excretion

Rats were housed in the general rat room and fed either high salt or high salt/fructose for 6–7 days. Then they were placed in metabolic cages while the appropriate diet was maintained. Urine was collected on dry ice for 24 h. Frozen urine samples were stored at −80°C until assayed. On the day of the assay most samples were thawed in warm water and measured directly. Some samples were diluted 1:1 with Tris‐buffered saline because the concentration of norepinephrine was out of range when measured without dilution. The composition of the Tris‐buffered saline used to dilute the samples was (in mmol/L): 132 NaCl, 40 Tris, 11.2 glucose, 4.2 KCl, 2 CaCl_2_, 0.7 MgCl_2_, pH 7.0. Norepinephrine was measured using continuous flow, fast scanning cyclic voltammetry by methods we previously reported (Chan et al., 2020). Urinary norepinephrine excretion was calculated based on the concentration of norepinephrine in an aliquot of the urine, the aliquot size, the total volume of urine excreted in 24 hrs and rat weight. Norepinephrine excretion was expressed as nmole/kg/day.

### 2.5 Statistics

Data are reported in the text as mean ± standard error of the mean, and plotted as either mean ± standard error of the mean or as box and whisker plots showing quartiles. Differences between means were analyzed using paired or unpaired *t*‐test as appropriate. *p* values <0.05 were considered significant.

## 3 RESULTS

**FIGURE 1:**
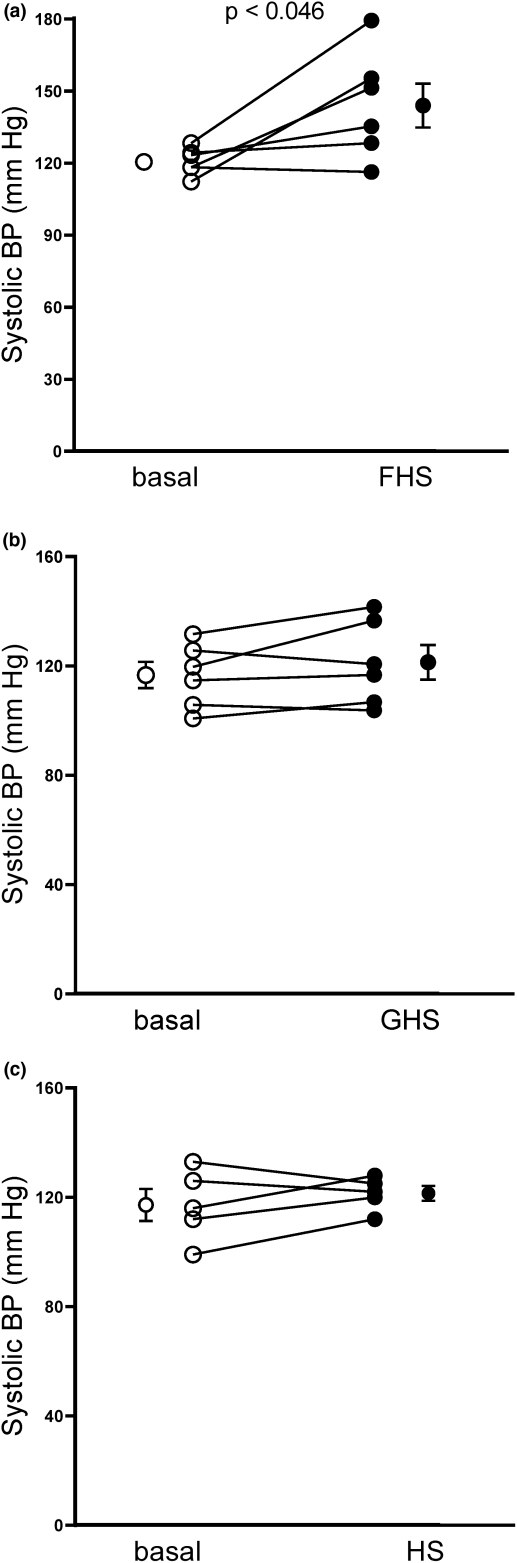
Effect of 7 days of fructose/high salt (FHS), glucose/high salt (GHS) and high salt alone (HS) on systolic blood pressure in male Sprague–Dawley rats as measured by plethysmography. (a) Effect of FHS (*n* = 6, *p* < 0.046). (b) Effect of GHS (*n* = 6, ns). C: Effect of HS (*n* = 5, ns). Basal: Blood pressure while on a standard rodent diet, before switching to either FHS, GHS or HS diets. Data were analyzed by paired *t*‐test.

We first measured the effect of fructose/high salt on systolic blood pressure compared to glucose/high salt as measured by plethysmography in male Sprague–Dawley rats (Figure 1). In the fructose/high salt group systolic blood pressure was 119 ± 5 when animals were on the standard rodent diet and 141 ± 4 mmHg (*n* = 6) after 7 days on the fructose/high salt diet, an increase of 22 ± 9 mmHg (Figure 1a; *p* < 0.046). In the glucose/high salt group blood pressure was 117 ± 5 mmHg when rats were on the standard rodent diet and 121 ± 6 mmHg (*n* = 6) after 7 days on the glucose/high salt diet (Figure 1b; Δ 5 ± 3 mmHg). Finally, we tested whether high salt alone altered systolic blood pressure. On the standard rodent diet, systolic blood pressure was 117 ± 6 mmHg and it was 121 ± 3 mmHg on high salt alone (Figure 1c; *n* = 5). Because the glucose/high salt diet and high salt alone did not increase blood pressure, male rats were fed high salt alone in subsequent protocols as the control.

**FIGURE 2:**
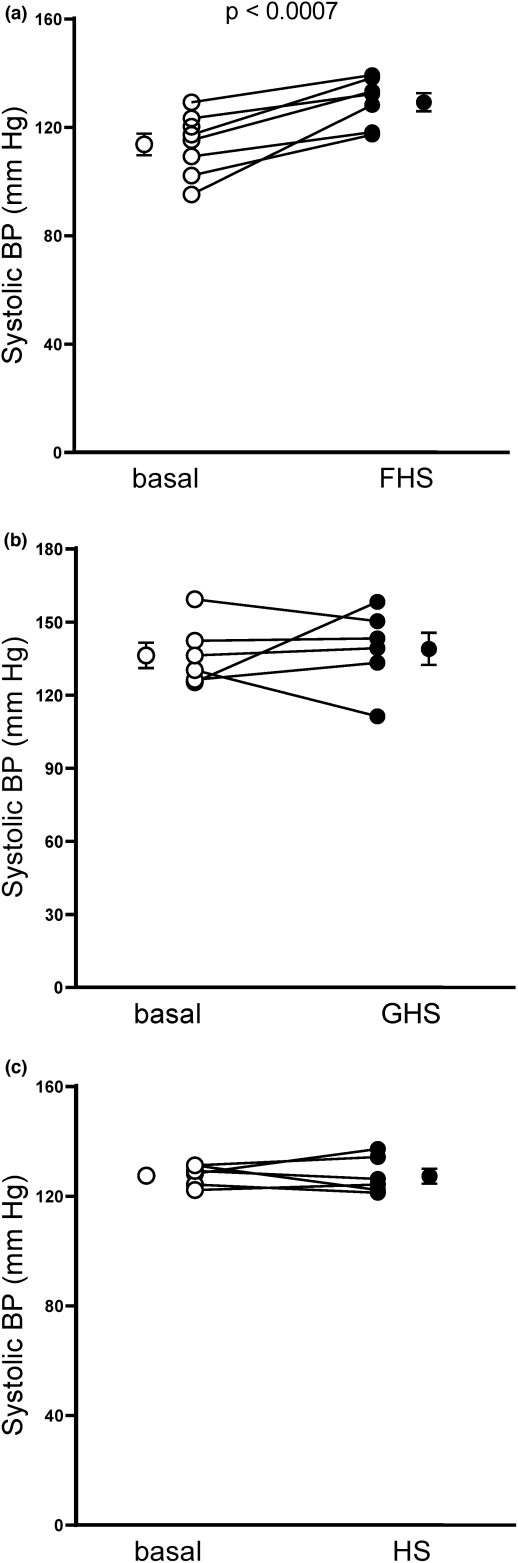
Effect of 7 days of fructose/high salt (FHS), glucose/high salt (GHS) and high salt alone (HS) on systolic blood pressure in female Sprague–Dawley rats as measured by plethysmography. (a) Effect of FHS (*n* = 8, *p* < 0.0007). (b) Effect of GHS (*n* = 6, ns). (c) Effect of HS (*n* = 6, ns). Basal: Blood pressure while on a standard rodent diet, before switching to either FHS, GHS or HS diets. Data were analyzed by paired *t*‐test.

We next studied systolic blood pressure in female Sprague–Dawley rats (Figure 2). Fructose/high salt increased systolic blood pressure from 114 ± 4 to 130 ± 3 mmHg in females (Figure 2a; *n* = 8; *p* < 0.0007). After 7 days on Glucose/high salt diet, systolic blood pressure on females was 139 ± 9 mmHg, not significantly different from the baseline (Figure 2b; *n* = 6). Interestingly the batch of female rats we fed Glucose/high salt, had a baseline systolic blood pressure of 136 ± 7 mmHg while on standard rodent diet. Finally, we tested whether high salt alone altered systolic blood pressure. On the standard rodent diet systolic blood pressure was 128 ± 2 mmHg and it was 127 ± 3 mmHg after 7 days on the high salt diet, not significantly different (Figure 2c; *n* = 6). Because the glucose/high salt diet and high salt alone did not increase blood pressure, female rats were fed high salt alone in subsequent protocols as the control.

**FIGURE 3:**
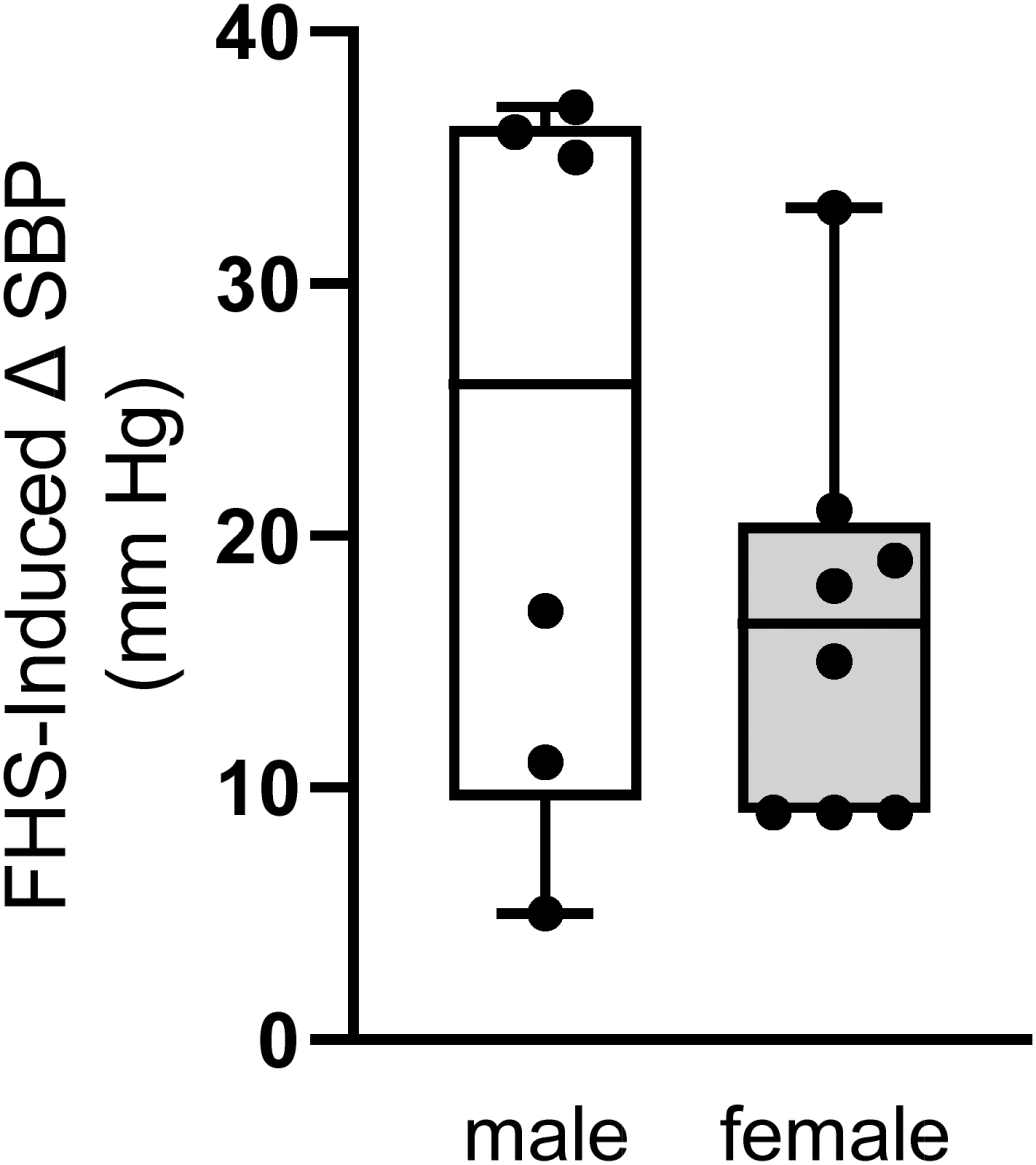
Increase in systolic blood pressure in male and female Sprague–Dawley rats caused by fructose/high salt as measured by plethysmography. There were no significant differences between males (*n* = 6) and females (*n* = 8). Data were analyzed by unpaired *t*‐test.

To study whether there were sex differences in the response of systolic blood pressure to the fructose/high salt diet, we compared the change in systolic blood pressures in male and female rats (Figure 3). The data for female rats is from Figure 2a. Fructose/high salt increased systolic blood pressure by 24 ± 6 (*n* = 6; *p* < 0.01) and 16 ± 3 mmHg (*n* = 8; *p* < 0.001) in males and females, respectively. These values were not significantly different.

In all of the above experiments, blood pressure was measured by plethysmography, which is moderately stressful for the rats. Stress has been suggested to be important in fructose‐induced hypertension due to activation of sympathetic nerves (Madero et al., 2011) but this has not been directly tested. Thus, we next tested whether measurements of blood pressure by plethysmography and telemetry differed, and whether that difference was due to stress in male Sprague–Dawley rats.

**FIGURE 4:**
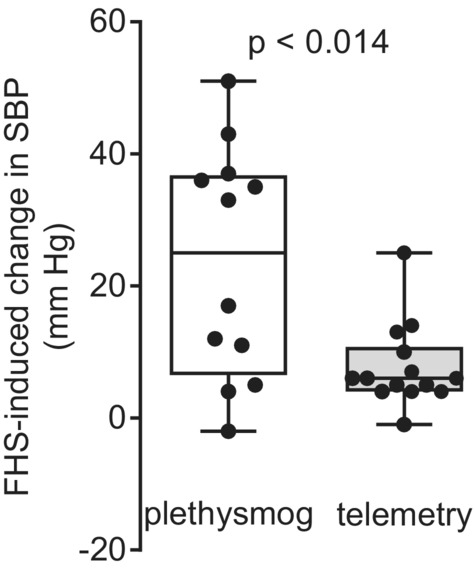
Fructose/high salt (FHS)‐induced change in systolic blood pressure (SBP) in male Sprague–Dawley rats as measured by plethysmography (plethysmog) and telemetry. The increase in systolic blood pressure was greater when measured by plethysmography (*n* = 12) compared to telemetry (*n* = 14) as analyzed by unpaired *t*‐test (*p* < 0.004).

The systolic blood pressures measured by plethysmography (moderate stress) in male rats from the combined data in Figures 1 and 3 show that fructose/high salt increased blood pressure by 24 ± 5 mmHg (*n* = 12). In contrast, when blood pressure was measured by telemetry allowing the rats to remain undisturbed in their cages (low stress), fructose/high salt raised blood pressure by only 8 ± 2 mmHg (*n* = 14). This increase was significantly lower than the increase measured by plethysmography (Figure 4; *p* < 0.014).

**FIGURE 5:**
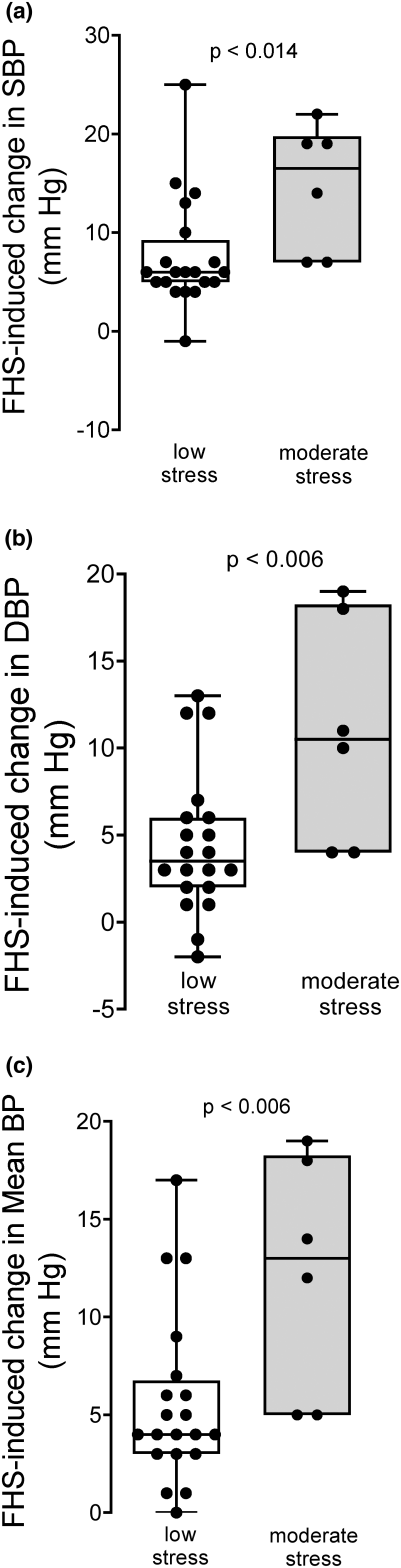
Change in blood pressure caused by 7 days of a fructose/high salt diet (FHS) in male Sprague–Dawley rats subjected to low (*n* = 20) and moderate stress (*n* = 6) as measured by telemetry. (a) Systolic blood pressure (SBP), (b) Diastolic blood pressure (DBP), and (c) Mean arterial pressure (mean BP). Data were analyzed by unpaired *t*‐test.

To test whether the difference seen in Figure 4 was due to stress per se, we measured the effect of fructose/high salt on systolic, diastolic and mean blood pressure by telemetry under low and moderate stress conditions as described in the Methods Section. In these experiments, stress was the independent variable. Under low stress conditions, fructose/high salt increased systolic blood pressure in males by 8 ± 1 mmHg (*n* = 20). In contrast, the increase in systolic blood pressure caused by fructose/high salt under moderate stress conditions in males was 15 ± 3 mmHg (*n* = 6), significantly greater than the increase caused when systolic blood pressure was measured under low stress conditions (Figure 5a; *p* < 0.014). Under low stress conditions, fructose/high salt increased diastolic blood pressure in males by 4 ± 1 mmHg (*n* = 20). In contrast, the increase in diastolic blood pressure caused by fructose/high salt under moderate stress conditions in males was 11 ± 3 mmHg (*n* = 6), significantly greater than the increase caused when diastolic blood pressure was measured under low stress conditions (Figure 5b; *p* < 0.006). Under low stress conditions, fructose/high salt increased mean blood pressure in males by 6 ± 1 mmHg (*n* = 20). In contrast, the increase in mean blood pressure caused by fructose/high salt under moderate stress conditions in males was 12 ± 3 mmHg (*n* = 6), significantly greater than the increase caused when mean blood pressure was measured under low stress conditions (Figure 5c; *p* < 0.006).

**FIGURE 6:**
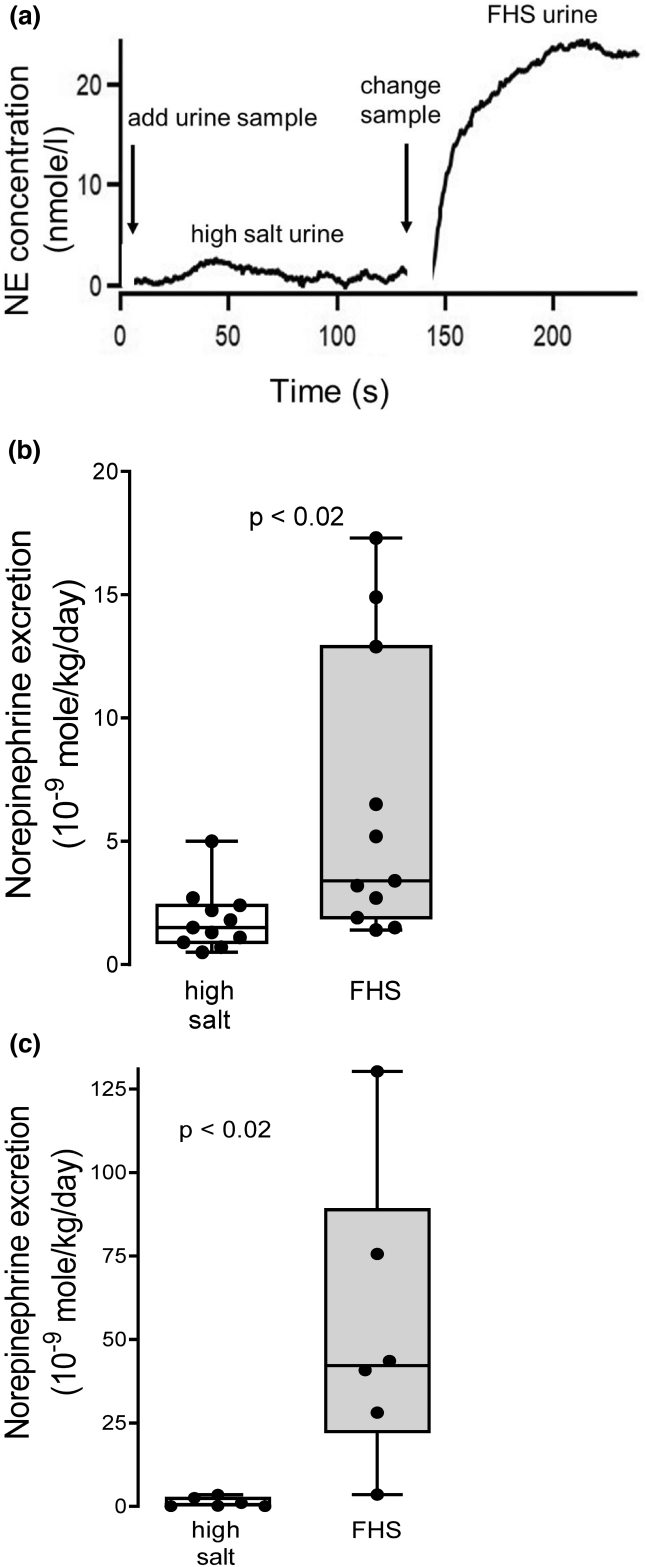
Effects of fructose/high salt on urinary norepinephrine (NE) excretion. (a) Representative trace from continuous flow, fast scanning cyclic voltammetry of urine from a rat on high salt and then fructose/high salt. (b) Urinary NE excretion in male animals fed fructose/high salt (FHS; *n* = 11) compared to high salt alone (*n* = 11). (c) Urinary NE excretion in female animals fed fructose/high salt (FHS; *n* = 6) compared to high salt alone (*n* = 6). Urinary NE concentration in 24 h urine was normalized by rat weight and expressed as nmole/kg/day. Data were analyzed by unpaired *t*‐test.

Since fructose‐induced increases in systolic blood pressure in males correlated with stress and this diet increased systolic blood pressure to a similar extent in females, we measured urinary norepinephrine excretion by male and female Sprague–Dawley rats under moderate stress conditions, i.e., conditions mimicking measurement of blood pressure by plethysmography (Figure 6). Urinary norepinephrine excretion primarily reflects activation of renal sympathetic nerves (Lappe et al., 1982; Osborn & Foss, 2017). Male rats on fructose/high salt (*n* = 11) excreted 6.4 ± 1.7 nmole/kg/day of norepinephrine. In contrast, male controls (*n* = 11) excreted 1.8 ± 0.4 nmole/Kg/day; a significantly lower amount (Figure 6b; *p* < 0.02). Norepinephrine excretion was also significantly greater (Figure 6c; *n* = 6; *p* < 0.02) in female rats fed fructose/high salt: 54 ± 18 nmole/kg/day, compared to female rats fed high salt alone: 1.2 ± 0.6 nmole/kg/day.

**FIGURE 7:**
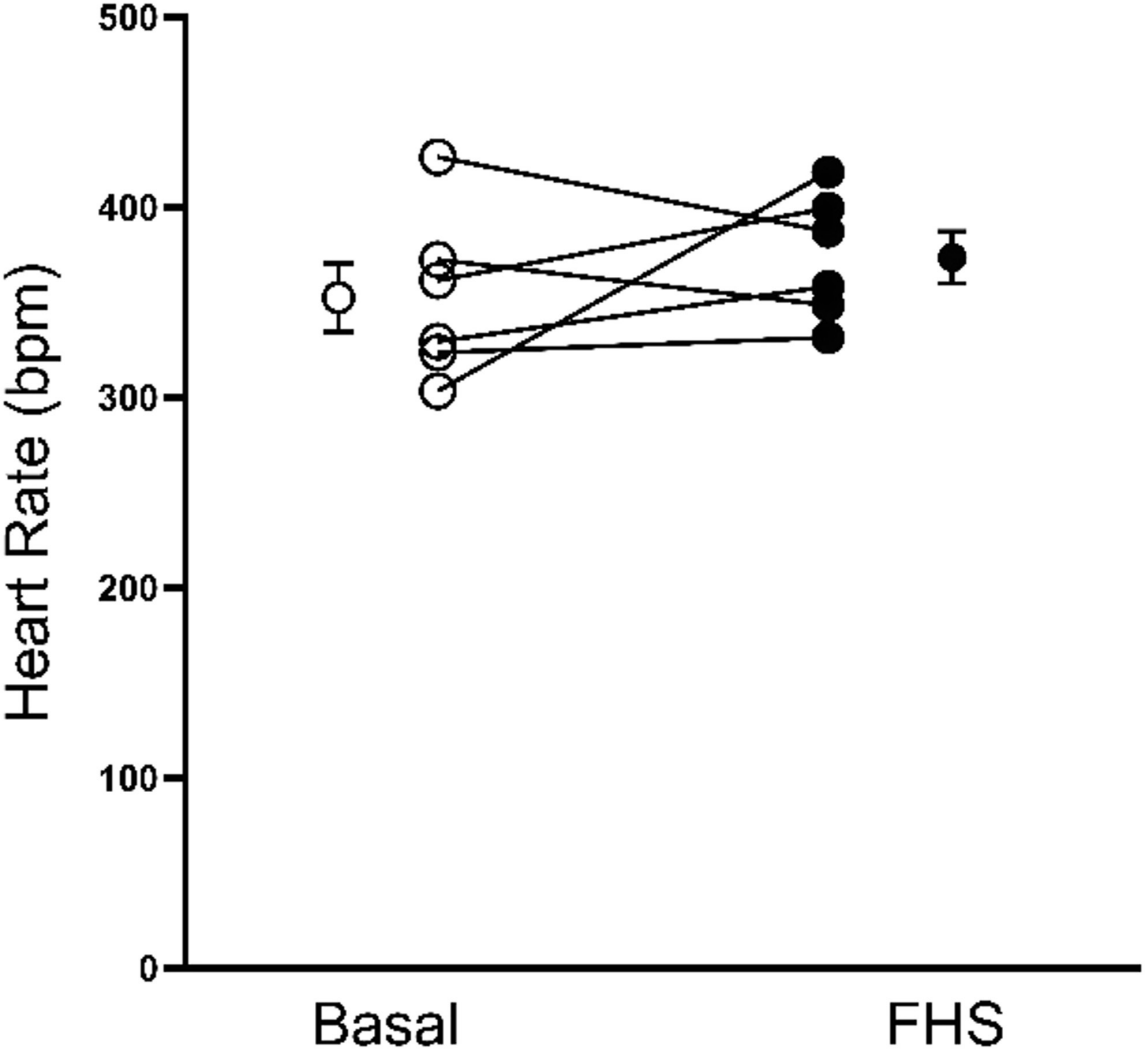
Effect of 7 days of a fructose/high salt diet (FHS) on heart rate in male Sprague–Dawley rats. Basal: Blood pressure while on a standard rodent diet, before switching to FHS. FSH: After 7–8 days in FSH diet. Data were analyzed by paired *t*‐test (*n* = 6; ns). “Bpm”, Beats per minute.

Finally, to test whether the fructose/high salt diet increased sympathetic regulation of the heart, we measured the effect of the fructose/high salt diet on heart rate in males (Figure 7). Basal heart rate in male rats was 352 ± 18 beats/min. Seven days after rats were on the fructose/high salt diet it was 374 ± 14 beats/min, not significantly different (*n* = 6).

## 4 DISCUSSION

We hypothesized that females are resistant to the salt‐sensitive hypertension caused by low amounts of dietary fructose compared to males and the magnitude of the increase in blood pressure depends, in part, on amplification of the stress response of renal sympathetic nerves. We found that moderate amounts of fructose in combination with a high salt diet increased blood pressure by 15–20 mmHg while the same amount of glucose with a high salt diet, or high salt alone had no significant effect in 7 days. These data show that fructose induces salt‐sensitive hypertension. The sensitivity of blood pressure to salt induced by moderate fructose consumption was similar in male and female rats. In addition, the combination of fructose and high salt increased blood pressure when rats were subjected to moderate stress, but the effect was significantly diminished when stress was low. Finally, consumption of fructose in combination with high salt increased urinary norepinephrine excretion in both sexes compared to high salt alone.

Here we found that a diet of 20% fructose in the drinking water and 4% NaCl caused hypertension within 7 days in male and female rats. This amount of fructose is comparable to that consumed by more than 17 million Americans. The data reported here are consistent with reports we (Cabral et al., 2014; Gonzalez‐Vicente et al., 2018) and others (Gordish et al., 2017; Zenner et al., 2018) have previously published. However, in our earlier studies we did not investigate whether fructose induces salt‐sensitive hypertension in female rats. One might expect that the blood pressure response in male and female rats would differ because fructose‐induced hypertension has been shown to depend on an activated RAS (Farah et al., 2007; Navarro‐Cid et al., 1995) and females are more resistant to the pro‐hypertensive actions of Ang II than are males (Brown et al., 2012; Gillis & Sullivan, 2016; Sampson et al., 2008; Shukri et al., 2018; Xue et al., 2005; Zimmerman & Sullivan, 2013). In contrast to this supposition, we found that fructose/high salt increased blood pressure to a similar extent in males and females.

Our results differ from those of Galipeau and coworkers (Galipeau et al., 2002). These investigators reported that female rats were protected from fructose‐induced changes in blood pressure. The explanation for the disparate results likely lies in the models. Our rats consumed 20% fructose in the drinking water plus high salt for 1 week, a model that does not cause changes in body weight, glycaemia or insulinaemia (Gonzalez‐Vicente et al., 2018), while Galipeau et al. (2002) used a well‐established model of metabolic syndrome for 9 weeks. In the model used by Galipeau et al (2002), rats are fed a diet in which basically all carbohydrates are replaced by fructose to a content of 60% by weight, representing 60%–70% of the caloric intake. In contrast, only 20%–30% of their caloric intake in our model is fructose, which more closely reflects fructose consumption by a large number of Americans.

The effects of fructose on blood pressure have been studied for many years, and yet the literature is inconsistent (Madero et al., 2011). There are many reports (Abdulla et al., 2012; Dai & McNeill, 1995; Gordish et al., 2017; Miatello et al., 2005; Soncrant et al., 2018; Vasdev et al., 2010; Zenner et al., 2018) including our own (Cabral et al., 2014; Gonzalez‐Vicente et al., 2018) showing that low to moderate amounts of fructose ranging between 10% and 30% of caloric intake increase blood pressure, and/or cause salt‐sensitive hypertension. This can occur without increases in plasma glucose, insulin resistance or plasma lipids (Gonzalez‐Vicente et al., 2017, 2018; Gordish et al., 2017). Previously, we postulated that the effect of fructose on blood pressure was dependent on at least three variables: amount of fructose in the diet; amount of salt in the diet; and time (Gonzalez‐Vicente et al., 2018). However, the method of blood pressure measurement may also affect the results. Thus, we compared plethysmography versus telemetry as means of recording the effects of dietary fructose and salt on blood pressure. We found that when measured by plethysmography, fructose/high salt increased blood pressure about twice as much than when blood pressure was measured by telemetry without disturbing the rats. We concluded the data were, in fact, accurate and sought a physiological explanation for these results.

Plethysmography causes stress in rats in a manner that telemetric measurement of blood pressure does not. Specifically, rats have to be transferred to a different room, removed from their cages, restrained, and subjected to heat and tactile stimuli. Additionally, the magnitude of the increase in blood pressure caused by angiotensin II has been reported to depend on the amount of stress induced by the method of measurement (King et al., 2006). Although the literature suggests that stress is an important factor (Madero et al., 2011), this has not been directly tested. Here we found that fructose‐induced salt‐sensitivity of blood pressure was exacerbated by a moderate amount of stress as is caused by measuring blood pressure by plethysmography. These findings were verified by measuring blood pressure using telemetry and subjecting rats to low and moderate amounts of stress; the latter similar to that caused by recording pressure by plethysmography.

These experiments were designed to evaluate the effect of stress, as an independent variable, on blood pressure. The moderate stress received by the fructose/high‐salt group was of the same magnitude as the one received by the glucose/high‐salt and high‐salt alone groups in Figure 1b,c. As glucose/high‐salt or high‐salt alone showed no increase in blood pressure, there was no rationale to evaluate the effect of reducing stress in the blood pressure response to diet. Thus, we did not test the effect of stress as an independent variable on blood pressure in rats fed glucose/high salt or high salt alone.

Our data suggest that stress contributes to fructose‐induced salt‐sensitive hypertension. To further investigate this effect, we measured urinary norepinephrine excretion in rats on high salt alone and fructose/high salt that had been subjected to a moderate amount of stress. We found that rats on fructose/high salt excreted significantly more norepinephrine than rats on salt alone. These data indicate that dietary fructose amplifies the sympathetic response, especially of renal nerves, to stress.

To investigate whether a fructose/high salt diet alters sympathetic innervation to all organs or the kidneys selectively, we measured heart rates in male rats fed fructose/high salt. We found that the fructose/high salt diet did not significantly alter heart rate. As fructose/high salt increased systolic blood pressure, one would have expected heart rate to decrease all things being equal; this was not the case. There are several possible explanations for the data. First is that the increase in systolic blood pressure was not sufficient to reduce heart rate. Second, activation of cardiac sympathetic nerves may have prevented the decrease. Finally, a decrease in parasympathetic activity caused by fructose/high salt may have augmented heart rate even in the face of an increase in blood pressure. The latter possibilities are supported by data showing that increases in plasma fructose alter brain activity and metabolism (Cigliano et al., 2018; Cisternas et al., 2015; Farah et al., 2006; Purnell et al., 2011). While resolution of this issue deserves further investigation, it is well beyond the scope of the current work. We did not study the effect of fructose/high salt on heart rate in female rats because the increase in blood pressure and increase in norepinephrine excretion caused by the fructose/high salt diet was not significantly different between males and females.

Our results show an important role for stress and sympathetic response in fructose‐induced salt‐sensitive hypertension. They are supported by data showing that renal denervation blunts the elevation in blood pressure (Soncrant et al., 2018). However, in that study, all rats were subjected to the same level of stress and stress was not an independent variable. Others have also reported that fructose‐induced changes in blood pressure correlated with stress (Madero et al., 2011) and urinary excretion of norepinephrine (Kamide et al., 2002) but did not directly test this hypothesis by using stress as an independent variable. Our results may explain why some investigators have found that dietary fructose is detrimental while others have not in terms of blood pressure and renal function in the absence of metabolic syndrome.

The regulation of renal function by sympathetic nerves is complex. Sympathetic nerves innervate the renal vasculature and many nephron segments including proximal tubules, thick ascending limbs and collecting ducts (Osborn & Foss, 2017). Additionally, at least five different adrenergic receptors are expressed in the kidney (Meister et al., 1994; Michel et al., 1993). Some tissues express more than one receptor (Plato, 2001; Snavely & Insel, 1982). Study of the specific receptor(s) involved in fructose‐induced salt‐sensitive hypertension is further complicated by the fact that use of receptor antagonists would have systemic effects outside the kidneys, which would seriously confound interpretation of the data. Although it is an important question as to which renal adrenergic receptor(s) are involved in the prohypertensive/salt‐retaining effects of dietary fructose, the experiments required for such a study are well beyond the scope of the present work.

Our conclusion that fructose‐induced salt‐sensitive hypertension is dependent on four variables: amount of fructose in the diet; amount of salt in the diet; the duration of special diets; and now stress or sympathetic tone is not unique to this model. Furthermore, the new finding that stress is important should serve warning that there are likely many variables that contribute to the development of hypertension that are poorly understood, and thus poorly controlled.

The literature supports the hypothesis that both activation of the RAS and sympathetic nerves are important for the development of fructose‐induced hypertension. However, it is currently unclear which comes first because no studies address this issue. Additionally, increased sympathetic tone has been shown to stimulate renin release (DiBona, 2000) and elevated angiotensin II enhances sympathetic nerve activity (DiBona, 2003; Rossi et al., 2010).

In summary, we have found that salt‐sensitive hypertension caused by dietary fructose afflicts males and females similarly and the magnitude of the increase in blood pressure is related to the level of stress. Given that the amount of fructose used in this study is similar to that consumed by people, our results suggest a cause for the prevalence of hypertension in general, and salt‐sensitivity in particular, in the US population.

## AUTHOR CONTRIBUTIONS

Autumn Brostek, Corey Smith, Jeffrey L. Garvin and Agustin Gonzalez‐Vicente conceived and designed research; Autumn Brostek, Nancy J. Hong, Ronghao Zhang, Beau R. Forester, Lauren E. Barmore, Lindsey Kaydo, and Nicholas Kluge performed experiments and collected data; Autumn Brostek, Nancy J. Hong, Ronghao Zhang, Beau R. Forester, Lauren E. Barmore, Corey Smith, and Jeffrey L. Garvin analyzed data; Autumn Brostek, Jeffrey L. Garvin and Agustin Gonzalez‐Vicente interpreted results; Autumn Brostek, Jeffrey L. Garvin and Agustin Gonzalez‐Vicente prepared the manuscript and figures with input from all authors. All authors provided critical feedback and helped shape the research, analysis and manuscript. All authors approved the final version of the manuscript.

## FUNDING INFORMATION

This work was supported in part by grants from the National Institutes of Health, National Heart, Lung and Blood Institute HL120853 to J.L.G. and National Institute of Biomedical Imaging and Bioengineering U01EB025138 to C.S. A.G‐V. was supported in part by DK007470 during this work.

## CONFLICT OF INTEREST

The authors have no conflict of interest to declare.

## References

[phy215489-bib-0001] Abdulla, M. H. , Sattar, M. A. , Johns, E. J. , Abdullah, N. A. , Hye Khan, M. A. , & Rathore, H. A. (2012). High‐fructose feeding impacts on the adrenergic control of renal haemodynamics in the rat. The British Journal of Nutrition, 107, 218–228. 21733307 10.1017/S0007114511002716

[phy215489-bib-0002] Briefel, R. R. , & Johnson, C. L. (2004). Secular trends in dietary intake in the United States. Annual Review of Nutrition, 24, 401–431. 10.1146/annurev.nutr.23.011702.073349 15189126

[phy215489-bib-0003] Brown, I. J. , Stamler, J. , Van Horn, L. , Robertson, C. E. , Chan, Q. , Dyer, A. R. , Huang, C. C. , Rodriguez, B. L. , Zhao, L. , Daviglus, M. L. , Ueshima, H. , & Elliott, P. (2011). International study of MM, blood pressure research G. sugar‐sweetened beverage, sugar intake of individuals, and their blood pressure: International study of macro/micronutrients and blood pressure. Hypertension (Dallas, TX: 1979), 57, 695–701. 10.1161/HYPERTENSIONAHA.110.165456 PMC3086758 21357284

[phy215489-bib-0004] Brown, R. D. , Hilliard, L. M. , Head, G. A. , Jones, E. S. , Widdop, R. E. , & Denton, K. M. (2012). Sex differences in the pressor and tubuloglomerular feedback response to angiotensin II. Hypertension (Dallas, TX: 1979), 59, 129–135. 10.1161/HYPERTENSIONAHA.111.178715 22124434

[phy215489-bib-0005] Cabral, P. D. , Hong, N. J. , Hye Khan, M. A. , Ortiz, P. A. , Beierwaltes, W. H. , Imig, J. D. , & Garvin, J. L. (2014). Fructose stimulates Na/H exchange activity and sensitizes the proximal tubule to angiotensin II. Hypertension (Dallas, TX: 1979), 63, e68–e73. 10.1161/HYPERTENSIONAHA.113.02564 24379189

[phy215489-bib-0006] Chan, S. A. , Vaseghi, M. , Kluge, N. , Shivkumar, K. , Ardell, J. L. , & Smith, C. (2020). Fast in vivo detection of myocardial norepinephrine levels in the beating porcine heart. American Journal of Physiology Heart and Circulatory Physiology, 318, H1091–H1099. 32216617 10.1152/ajpheart.00574.2019 PMC7346543

[phy215489-bib-0007] Cigliano, L. , Spagnuolo, M. S. , Crescenzo, R. , Cancelliere, R. , Iannotta, L. , Mazzoli, A. , Liverini, G. , & Iossa, S. (2018). Short‐term fructose feeding induces inflammation and oxidative stress in the hippocampus of young and adult rats. Molecular Neurobiology, 55, 2869–2883. 28455700 10.1007/s12035-017-0518-2

[phy215489-bib-0008] Cisternas, P. , Salazar, P. , Serrano, F. G. , Montecinos‐Oliva, C. , Arredondo, S. B. , Varela‐Nallar, L. , Barja, S. , Vio, C. P. , Gomez‐Pinilla, F. , & Inestrosa, N. C. (2015). Fructose consumption reduces hippocampal synaptic plasticity underlying cognitive performance. Biochimica et Biophysica Acta, 1852, 2379–2390. 26300486 10.1016/j.bbadis.2015.08.016 PMC5369608

[phy215489-bib-0009] Dai, S. , & McNeill, J. H. (1995). Fructose‐induced hypertension in rats is concentration‐ and duration‐dependent. Journal of Pharmacological and Toxicological Methods, 33, 101–107. 7766916 10.1016/1056-8719(94)00063-a

[phy215489-bib-0010] Di Verniero, C. A. , Silberman, E. A. , Mayer, M. A. , Opezzo, J. A. , Taira, C. A. , & Höcht, C. (2008). In vitro and in vivo pharmacodynamic properties of metoprolol in fructose‐fed hypertensive rats. Journal of Cardiovascular Pharmacology, 51, 532–541. 18475202 10.1097/FJC.0b013e3181730306

[phy215489-bib-0011] DiBona, G. F. (2000). Nervous kidney. Interaction between renal sympathetic nerves and the renin‐angiotensin system in the control of renal function. Hypertension (Dallas, TX: 1979), 36, 1083–1088. 10.1161/01.hyp.36.6.1083 11116129

[phy215489-bib-0012] DiBona, G. F. (2003). Central angiotensin modulation of baroreflex control of renal sympathetic nerve activity in the rat: Influence of dietary sodium. Acta Physiologica Scandinavica, 177, 285–289. 12608998 10.1046/j.1365-201X.2003.01074.x

[phy215489-bib-0013] El‐Bassossy, H. M. , & Shaltout, H. A. (2015). Allopurinol alleviates hypertension and proteinuria in high fructose, high salt and high fat induced model of metabolic syndrome. Translational Research, 165, 621–630. 25528722 10.1016/j.trsl.2014.11.008

[phy215489-bib-0014] Farah, V. , Elased, K. M. , Chen, Y. , Key, M. P. , Cunha, T. S. , Irigoyen, M. C. , & Morris, M. (2006). Nocturnal hypertension in mice consuming a high fructose diet. Autonomic Neuroscience, 130, 41–50. 16843071 10.1016/j.autneu.2006.05.006

[phy215489-bib-0015] Farah, V. , Elased, K. M. , & Morris, M. (2007). Genetic and dietary interactions: Role of angiotensin AT1a receptors in response to a high‐fructose diet. American Journal of Physiology Heart and Circulatory Physiology, 293, H1083–H1089. 17449556 10.1152/ajpheart.00106.2006

[phy215489-bib-0016] Galipeau, D. , Verma, S. , & McNeill, J. H. (2002). Female rats are protected against fructose‐induced changes in metabolism and blood pressure. American Journal of Physiology Heart and Circulatory Physiology, 283, H2478–H2484. 12427595 10.1152/ajpheart.00243.2002

[phy215489-bib-0017] Gersch, M. S. , Mu, W. , Cirillo, P. , Reungjui, S. , Zhang, L. , Roncal, C. , Sautin, Y. Y. , Johnson, R. J. , & Nakagawa, T. (2007). Fructose, but not dextrose, accelerates the progression of chronic kidney disease. American Journal of Physiology Renal Physiology, 293, F1256–F1261. 17670904 10.1152/ajprenal.00181.2007

[phy215489-bib-0018] Gillis, E. E. , & Sullivan, J. C. (2016). Sex differences in hypertension: Recent advances. Hypertension (Dallas, TX: 1979), 68, 1322–1327. 10.1161/HYPERTENSIONAHA.116.06602 PMC5159215 27777357

[phy215489-bib-0019] Gonzalez‐Vicente, A. , Cabral, P. D. , Hong, N. J. , Asirwatham, J. , Yang, N. , Berthiaume, J. M. , Dominici, F. P. , & Garvin, J. L. (2017). Dietary fructose enhances the ability of low concentrations of angiotensin II to stimulate proximal tubule Na(+) reabsorption. Nutrients, 9, 885. 10.3390/nu9080885 PMC5579678 28813008

[phy215489-bib-0020] Gonzalez‐Vicente, A. , Hong, N. J. , Yang, N. , Cabral, P. D. , Berthiaume, J. M. , Dominici, F. P. , & Garvin, J. L. (2018). Dietary fructose increases the sensitivity of proximal tubules to angiotensin II in rats fed high‐salt diets. Nutrients, 10(9), 1244. 10.3390/nu10091244 PMC6164674 30200571

[phy215489-bib-0021] Gordish, K. L. , Kassem, K. M. , Ortiz, P. A. , & Beierwaltes, W. H. (2017). Moderate (20%) fructose‐enriched diet stimulates salt‐sensitive hypertension with increased salt retention and decreased renal nitric oxide. Physiological Reports, 5(7), e13162. 28408634 10.14814/phy2.13162 PMC5392503

[phy215489-bib-0022] Hallfrisch, J. (1990). Metabolic effects of dietary fructose. The FASEB Journal, 4, 2652–2660. 2189777 10.1096/fasebj.4.9.2189777

[phy215489-bib-0023] Hong, N. J. , Gonzalez‐Vicente, A. , Saez, F. , & Garvin, J. L. (2021). Mechanisms of decreased tubular flow‐induced nitric oxide in dahl salt‐sensitive rat thick ascending limbs. American Journal of Physiology Renal Physiology, 321, F369–F377. 34308669 10.1152/ajprenal.00124.2021 PMC8530749

[phy215489-bib-0024] Hwang, I. S. , Ho, H. , Hoffman, B. B. , & Reaven, G. M. (1987). Fructose‐induced insulin resistance and hypertension in rats. Hypertension (Dallas, TX: 1979), 10, 512–516. 10.1161/01.hyp.10.5.512 3311990

[phy215489-bib-0025] Jalal, D. I. , Smits, G. , Johnson, R. J. , & Chonchol, M. (2010). Increased fructose associates with elevated blood pressure. Journal of the American Society of Nephrology, 21, 1543–1549. 20595676 10.1681/ASN.2009111111 PMC3013529

[phy215489-bib-0026] Kamide, K. , Rakugi, H. , Higaki, J. , Okamura, A. , Nagai, M. , Moriguchi, K. , Ohishi, M. , Satoh, N. , Tuck, M. L. , & Ogihara, T. (2002). The renin‐angiotensin and adrenergic nervous system in cardiac hypertrophy in fructose‐fed rats. American Journal of Hypertension, 15, 66–71. 11824863 10.1016/s0895-7061(01)02232-4

[phy215489-bib-0027] King, A. J. , Zorn, C. , & Fink, G. (2006). The hypertensive response to chronic low‐dose angiotensin II (ang II) is dependent on arterial pressure (AP) measurement method and salt intake. The FASEB Journal, 20(4), A754.

[phy215489-bib-0028] Komnenov, D. , Levanovich, P. E. , & Rossi, N. F. (2019). Hypertension associated with fructose and high salt: Renal and sympathetic mechanisms. Nutrients, 11(3), 569. 10.3390/nu11030569 PMC6472002 30866441

[phy215489-bib-0029] Lappe, R. W. , Henry, D. P. , & Willis, L. R. (1982). Contribution of renal sympathetic nerves to the urinary excretion of norepinephrine. Canadian Journal of Physiology and Pharmacology, 60, 1067–1072. 7127218 10.1139/y82-153

[phy215489-bib-0030] Madero, M. , Perez‐Pozo, S. E. , Jalal, D. , Johnson, R. J. , & Sanchez‐Lozada, L. G. (2011). Dietary fructose and hypertension. Current Hypertension Reports, 13, 29–35. 20957458 10.1007/s11906-010-0163-x

[phy215489-bib-0031] Marriott, B. P. , Cole, N. , & Lee, E. (2009). National estimates of dietary fructose intake increased from 1977 to 2004 in the United States. The Journal of Nutrition, 139, 1228S–1235S. 19403716 10.3945/jn.108.098277

[phy215489-bib-0032] Meister, B. , Dagerlind, A. , Nicholas, A. P. , & Hokfelt, T. (1994). Patterns of messenger RNA expression for adrenergic receptor subtypes in the rat kidney. The Journal of Pharmacology and Experimental Therapeutics, 268, 1605–1611. 8138972

[phy215489-bib-0033] Miatello, R. , Vazquez, M. , Renna, N. , Cruzado, M. , Zumino, A. P. , & Risler, N. (2005). Chronic administration of resveratrol prevents biochemical cardiovascular changes in fructose‐fed rats. American Journal of Hypertension, 18, 864–870. 15925749 10.1016/j.amjhyper.2004.12.012

[phy215489-bib-0034] Michel, M. C. , Siepmann, F. , Buscher, R. , Philipp, T. , & Brodde, O. E. (1993). Ontogenesis of sympathetic responsiveness in spontaneously hypertensive rats. I. Renal alpha 1‐, alpha 2‐, and beta‐adrenergic receptors and their signaling. Hypertension (Dallas, TX: 1979), 22, 169–177. 10.1161/01.hyp.22.2.169 8393427

[phy215489-bib-0035] Mills, K. T. , Stefanescu, A. , & He, J. (2020). The global epidemiology of hypertension. Nature Reviews Nephrology, 16, 223–237. 32024986 10.1038/s41581-019-0244-2 PMC7998524

[phy215489-bib-0036] Mozaffarian, D. , Benjamin, E. J. , Go, A. S. , Arnett, D. K. , Blaha, M. J. , Cushman, M. , Das, S. R. , de Ferranti, S. , Després, J. P. , Fullerton, H. J. , Howard, V. J. , Huffman, M. D. , Isasi, C. R. , Jiménez, M. C. , Judd, S. E. , Kissela, B. M. , Lichtman, J. H. , Lisabeth, L. D. , Liu, S. , … Turner, M. B. (2016). Executive summary: Heart disease and stroke statistics—2016 update: A report from the American Heart Association. Circulation, 133, 447–454. 26811276 10.1161/CIR.0000000000000366

[phy215489-bib-0037] Nakayama, T. , Kosugi, T. , Gersch, M. , Connor, T. , Sanchez‐Lozada, L. G. , Lanaspa, M. A. , Roncal, C. , Perez‐Pozo, S. E. , Johnson, R. J. , & Nakagawa, T. (2010). Dietary fructose causes tubulointerstitial injury in the normal rat kidney. American Journal of Physiology Renal Physiology, 298, F712–F720. 20071464 10.1152/ajprenal.00433.2009 PMC2838595

[phy215489-bib-0038] Navarro‐Cid, J. , Maeso, R. , Perez‐Vizcaino, F. , Cachofeiro, V. , Ruilope, L. M. , Tamargo, J. , & Lahera, V. (1995). Effects of losartan on blood pressure, metabolic alterations, and vascular reactivity in the fructose‐induced hypertensive rat. Hypertension (Dallas, TX: 1979), 26, 1074–1078. 10.1161/01.hyp.26.6.1074 7498971

[phy215489-bib-0039] Nguyen, S. , Choi, H. K. , Lustig, R. H. , & Hsu, C. Y. (2009). Sugar‐sweetened beverages, serum uric acid, and blood pressure in adolescents. The Journal of Pediatrics, 154, 807–813. 19375714 10.1016/j.jpeds.2009.01.015 PMC2727470

[phy215489-bib-0040] Nishimoto, Y. , Tomida, T. , Matsui, H. , Ito, T. , & Okumura, K. (2002). Decrease in renal medullary endothelial nitric oxide synthase of fructose‐fed, salt‐sensitive hypertensive rats. Hypertension (Dallas, TX: 1979), 40, 190–194. 10.1161/01.hyp.0000024267.71656.0d 12154112

[phy215489-bib-0041] Osborn, J. W. , & Foss, J. D. (2017). Renal nerves and long‐term control of arterial pressure. Comprehensive Physiology, 7, 263–320. 28333375 10.1002/cphy.c150047

[phy215489-bib-0042] Plato, C. F. (2001). Alpha‐2 and beta‐adrenergic receptors mediate NE's biphasic effects on rat thick ascending limb chloride flux. American Journal of Physiology Regulatory, Integrative and Comparative Physiology, 281, R979–R986. 11507016 10.1152/ajpregu.2001.281.3.R979

[phy215489-bib-0043] Purnell, J. Q. , Klopfenstein, B. A. , Stevens, A. A. , Havel, P. J. , Adams, S. H. , Dunn, T. N. , Krisky, C. , & Rooney, W. D. (2011). Brain functional magnetic resonance imaging response to glucose and fructose infusions in humans. Diabetes, Obesity & Metabolism, 13, 229–234. 10.1111/j.1463-1326.2010.01340.x 21205113

[phy215489-bib-0044] Rossi, N. F. , Maliszewska‐Scislo, M. , Chen, H. , Black, S. M. , Sharma, S. , Ravikov, R. , & Augustyniak, R. A. (2010). Neuronal nitric oxide synthase within paraventricular nucleus: Blood pressure and baroreflex in two‐kidney, one‐clip hypertensive rats. Experimental Physiology, 95, 845–857. 20494920 10.1113/expphysiol.2009.051789 PMC2905784

[phy215489-bib-0045] Sampson, A. K. , Moritz, K. M. , Jones, E. S. , Flower, R. L. , Widdop, R. E. , & Denton, K. M. (2008). Enhanced angiotensin II type 2 receptor mechanisms mediate decreases in arterial pressure attributable to chronic low‐dose angiotensin II in female rats. Hypertension (Dallas, TX: 1979), 52, 666–671. 10.1161/HYPERTENSIONAHA.108.114058 18711010

[phy215489-bib-0046] Sanchez‐Lozada, L. G. , Tapia, E. , Jimenez, A. , Bautista, P. , Cristobal, M. , Nepomuceno, T. , Soto, V. , Avila‐Casado, C. , Nakagawa, T. , Johnson, R. J. , Herrera‐Acosta, J. , & Franco, M. (2007). Fructose‐induced metabolic syndrome is associated with glomerular hypertension and renal microvascular damage in rats. American Journal of Physiology Renal Physiology, 292, F423–F429. 16940562 10.1152/ajprenal.00124.2006

[phy215489-bib-0047] Sechi, L. A. (1999). Mechanisms of insulin resistance in rat models of hypertension and their relationships with salt sensitivity. Journal of Hypertension, 17, 1229–1237. 10489099 10.1097/00004872-199917090-00001

[phy215489-bib-0048] Shukri, M. Z. , Tan, J. W. , Manosroi, W. , Pojoga, L. H. , Rivera, A. , Williams, J. S. , Seely, E. W. , Adler, G. K. , Jaffe, I. Z. , Karas, R. H. , Williams, G. H. , & Romero, J. R. (2018). Biological sex modulates the adrenal and blood pressure responses to angiotensin II. Hypertension (Dallas, TX: 1979), 71, 1083–1090. 10.1161/HYPERTENSIONAHA.117.11087 PMC6086591 29686001

[phy215489-bib-0049] Snavely, M. D. , & Insel, P. A. (1982). Characterization of alpha‐adrenergic receptor subtypes in the rat renal cortex. Differential regulation of alpha 1‐ and alpha 2‐adrenergic receptors by guanyl nucleotides and Na. Molecular Pharmacology, 22, 532–546. 6296651

[phy215489-bib-0050] Soncrant, T. , Komnenov, D. , Beierwaltes, W. H. , Chen, H. , Wu, M. , & Rossi, N. F. (2018). Bilateral renal cryodenervation decreases arterial pressure and improves insulin sensitivity in fructose‐fed Sprague‐Dawley rats. American Journal of Physiology Regulatory, Integrative and Comparative Physiology, 315, R529–R538. 29847164 10.1152/ajpregu.00020.2018

[phy215489-bib-0051] Valensi, P. (2005). Hypertension, single sugars and fatty acids. Journal of Human Hypertension, 19(Suppl 3), S5–S9. 16302008 10.1038/sj.jhh.1001954

[phy215489-bib-0052] Vasdev, S. , Gill, V. D. , Randell, E. , Han, Y. , & Gadag, V. (2010). Fructose and moderately high dietary salt‐induced hypertension: Prevention by a combination of N‐acetylcysteine and L‐arginine. Molecular and Cellular Biochemistry, 337, 9–16. 19806432 10.1007/s11010-009-0281-4

[phy215489-bib-0053] Verma, S. , Bhanot, S. , & McNeill, J. H. (1999). Sympathectomy prevents fructose‐induced hyperinsulinemia and hypertension. European Journal of Pharmacology, 373, R1–R4. 10414447 10.1016/s0014-2999(99)00301-5

[phy215489-bib-0054] Xue, B. , Pamidimukkala, J. , & Hay, M. (2005). Sex differences in the development of angiotensin II‐induced hypertension in conscious mice. American Journal of Physiology Heart and Circulatory Physiology, 288, H2177–H2184. 15626687 10.1152/ajpheart.00969.2004

[phy215489-bib-0055] Zenner, Z. P. , Gordish, K. L. , & Beierwaltes, W. H. (2018). Free radical scavenging reverses fructose‐induced salt‐sensitive hypertension. Integrated Blood Pressure Control, 11, 1–9. 29296095 10.2147/IBPC.S147674 PMC5741067

[phy215489-bib-0056] Zimmerman, M. A. , & Sullivan, J. C. (2013). Hypertension: What's sex got to do with it? Physiology (Bethesda, MD), 28, 234–244. 10.1152/physiol.00013.2013 PMC3742130 23817798

